# Living benthic foraminifera from cold-water coral ecosystems in the eastern Alboran Sea, Western Mediterranean

**DOI:** 10.1016/j.heliyon.2021.e07880

**Published:** 2021-08-27

**Authors:** Claudio Stalder, Akram ElKateb, Jorge E. Spangenberg, Loubna Terhzaz, Agostina Vertino, Silvia Spezzaferri

**Affiliations:** a Department of Geosciences, University of Fribourg, Chemin du Musée 6, CH–1700 Fribourg, Switzerland; b Institute of Earth Surface Dynamics, University of Lausanne, Building Géopolis, CH–1015 Lausanne, Switzerland; c ODYSSEE Research Group, Geosciences, Water and Environment Laboratory, Faculty of Sciences in Rabat, Mohammed V University in Rabat, BP 1014 Rabat, Morocco; d Department of Geology, Ghent University, B-9000, Gent, Belgium; e Department of Earth and Environmental Sciences, University of Milano-Bicocca, I-20126, Milan, Italy

**Keywords:** Living foraminifera, Cold-water corals, Alboran sea, Mediterranean

## Abstract

Benthic foraminifera (protists with biomineralized tests) coupled with geochemical proxies are used for the first time to characterize present oceanographic conditions occurring in cold-water coral ecosystems (CWC) in the eastern Alboran Sea (Brittlestar Ridge and Cablier Mound), western Mediterranean Sea. Quantitative data on living (stained) benthic foraminifera from 5 box cores retrieved during the MD194 cruise on the RV Marion Dufresne reveal that these organisms are more diverse in presence of corals, where more numerous ecological niches occur than they are in pelagic adjacent sediments. These data confirm that CWC can be considered as “diversity hotspots” also for benthic foraminifera.

Geochemical characterization shows that these sediments contain relatively fresh (labile) organic matter but also a reworked refractory component. In particular, the total organic carbon and the *δ*^13^C_org_ values suggest that some of the organic matter may be a mixture of marine and reworked particulate organic matter, compared to typical values from temperate phytoplankton. The *δ*^15^N of the organic fraction suggests that important atmospheric N_2_-fixation and degradation processes occur in the region.

Finally, our results show that a more effective advection of freshly exported particulate organic matter from the surface waters occur at the mound top rather than at the mound base or off-mound allowing some coral colonies to survive on the top of mounds in this region. The mud layer covering the coral rubble debris may suggest that the Brittlestar Ridge is today exposed to siltation preventing the growth of corals at the mound base or off-mound.

## 1 Introduction

Cold-water coral (CWC) ecosystems are “hotspots” of biodiversity offering diversified habitats to marine organisms (Freiwald et al., 2004; Mastrototaro et al., 2010; Henry and Roberts, 2017). These ecosystems preferentially occur on subvertical walls, overhangs and pre-existing topographic highs with sustained nutrient and food particle input and where intense bottom currents prevent corals to be smothered by sediment (e.g., White et al., 2005; Davies et al., 2008; Vertino et al., 2010). In optimal conditions CWCs can create extensive three-dimensional bioconstructions and contribute to the formation of CWC mounds (e.g., Roberts et al., 2009; Wienberg and Titschack 2017), reaching up to hundreds of meters in height (Mienis et al., 2007). The geographical distribution of CWC bioconstructions, mainly concentrated along continental margins, is controlled by chemo-physical properties of the surrounding water masses, such as temperature, density and dissolved oxygen content (Dullo et al., 2008; Davies et al., 2008; Freiwald et al., 2009; Roberts et al., 2009). As hotspot of biodiversity, these ecosystems are presently a main concern for the scientific community for their sensitivity in changing environmental conditions. Moreover, they play an important role in the total carbonate budget and in the regulation of atmospheric CO_2_ on Earth (e.g., Lindberg and Mienert, 2005; Roberts et al., 2006; Movilla et al., 2014; Titschack et al., 2016).

Living solitary CWC species, such as *Desmophyllum dianthus* and *Caryophyllia calveri,* are very common throughout the Mediterranean (e.g., Zibrowius, 1980; Chimienti et al., 2019). However, CWCs including living colonial corals (mostly *Madrepora oculata* and *Desmophyllum pertusum* (syn. *Lophelia pertusa,* following Addamo et al., 2016) are patchy and occur in specific areas of this basin: on the coral mounds of the Alboran and Ionian Sea (Hebbeln et al., 2009; Corselli, 2010; Fink et al., 2013; Savini et al., 2014; Corbera et al., 2019), along flanks of canyons in the Gulf of Lions (Orejas et al., 2009; Gori et al., 2013), on the Ligurian margin (Tunesi et al., 2001; Fanelli et al., 2017), in the Strait of Sicily, Adriatic Sea and Sardinia Channel (Freiwald et al., 2009; Sanfilippo et al., 2013; Angeletti et al., 2014: D'Onghia et al., 2015; Taviani et al., 2017), and in the Aegean Sea (Zibrowius, 1980; Vafidis et al., 1997). Following Addamo et al. (2016), we use *Desmophyllum pertusum* to indicate also the branching CWC species known in the literature as *Lophelia pertusa.*

The Melilla Mound Field (MMF) CWC in the Alboran Sea was discovered in 2007 (RV Experides cruise - Comas and Pinheiro, 2007) and was first cored in 2008 and 2009 (Hebbeln et al., 2009). A preliminary research (Fink et al., 2013) revealed the presence of patchy living scleractinian coral colonies.

Microfaunal assemblages (e.g., benthic foraminifera and ostracods) can provide a very important proxy to understand CWC dynamics, from their nucleation, growth and decline (Margreth et al., 2009; Malinverno et al., 2010; Stalder et al., 2014, 2015; Pirkenseer et al., 2018; Fentimen et al., 2018). However, no studies on living benthic foraminifera assemblages associated to CWC ecosystems have been conducted so far in the Alboran Sea.

During the Cruise MD194 in 2013 (Mediterranean Gateway) five box cores were recovered in the MMF. Living (stained) benthic foraminifera and total organic carbon (TOC), organic carbon and nitrogen isotopes (*δ*^13^C_org_ and *δ*^15^N_org_) analyses were performed on surface sediments from these box cores with the aim of obtaining insights on: 1) the living benthic foraminiferal assemblages; 2) how benthic foraminiferal assemblages vary with respect to the framework-builders and associated neighbouring coral rubble and 3) the processes presently occurring at the sea floor in CWCs in the Alboran Sea.

## 2 Geological and oceanographic settings

**Figure 1:**
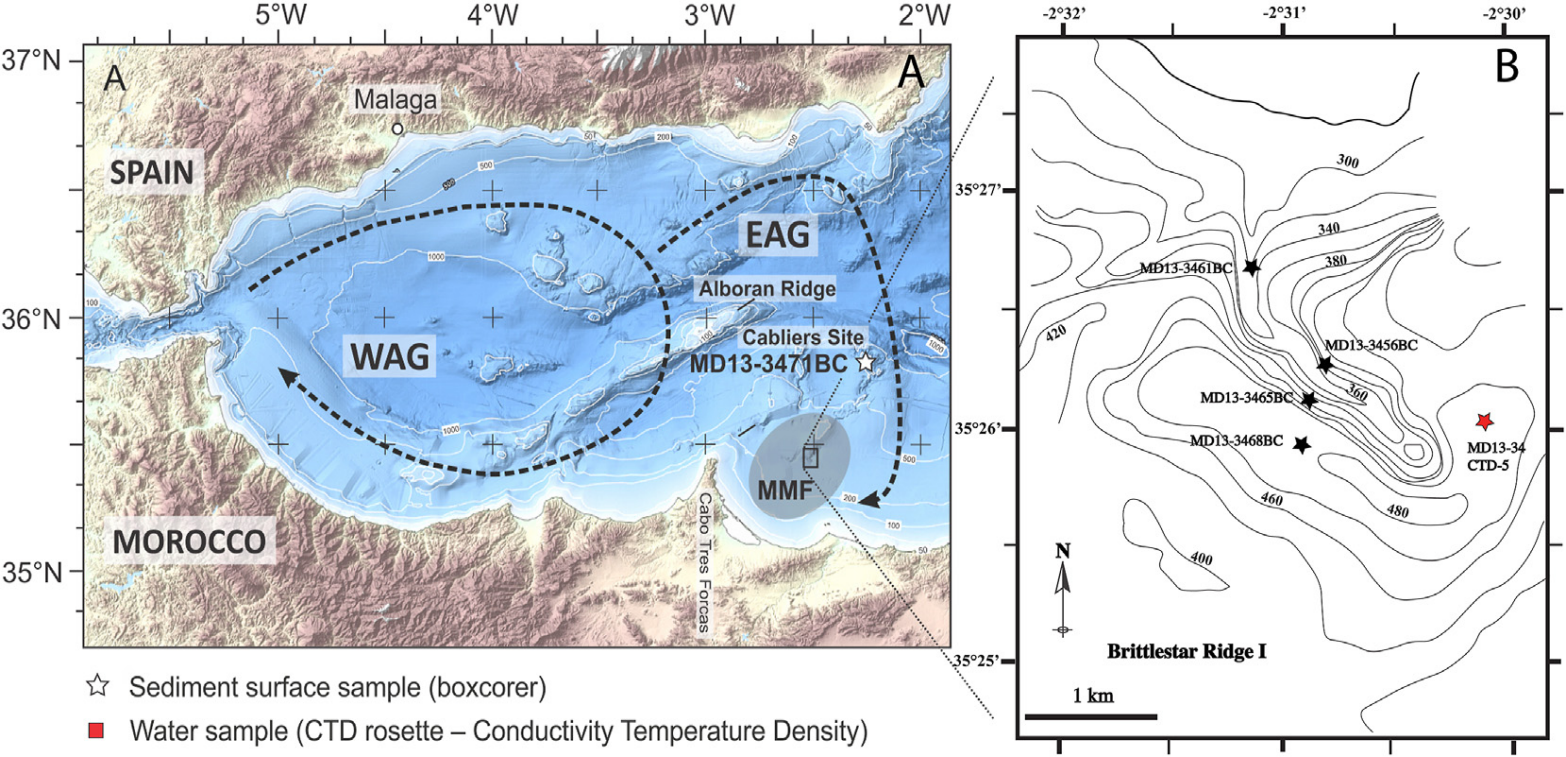
A. Location map of the study area in the Eastern Alboran Sea. The circulation of surface water in the Eastern and Western Alboran Gyres is also shown (modified after EMODnet Bathymetry Consortium, 2018). B. Schematic bathymetric map of the Brittlestar Ridge I in the MMF showing the position of the box cores and the CTD cast.

The MMF is located in the southern part of the Alboran Sea (western Mediterranean), east to the Cape Tres Forcas (Figure 1A). Clusters of CWC mounds and ridges occur in the MMF, covering a surface of ~100 km^2^ at 250–600 m water depth (Comas and Pinheiro, 2007; Lo Iacono et al., 2014). The size of these structures ranges from 48 m to 476 m in diameter, their length is up to 649 m and they can reach height up to 48 m. One to 12 m thick fine-grained sediment veneers bury some of these mounds (Lo Iacono et al., 2014). The "Banc de Provençaux" is located in the northern sector of the MMF, in a water depth of 200 m. Video surveys have revealed that this bank is characterized by the presence of elongated ridges hosting generally dead CWCs (Comas et al., 2009; Hebbeln et al., 2009) and that living corals colonies are only present on their tops (Hebbeln et al., 2009).

The N–S oriented Cabliers Bank (Ammar et al., 2007) of volcanic origin is located north to the MMF. It is 6 km wide and 12 km long is characterized by dense living CWCs (Lo Iacono et al., 2014; Corbera et al., 2019) (Figure 1A).

The flows of water masses in the Alboran Sea are modulated by the exchanges with the Atlantic Ocean (e.g., van Rooji et al., 2013; Stalder et al., 2018). Modified Atlantic Water (MAW) occurs between 0 and 200 m of water depth. They initially display a salinity around <36.5 but they become gradually more saline in function of their residence time and mixing with the Mediterranean water (Font et al., 1988). Flowing from the Atlantic to the Mediterranean the MAV form a jet that produces two anticyclonic gyres in the Western (WAG) and Eastern (EAG) Alboran basins, respectively (La Violette, 1986) (Figure 1A). The two gyres are relatively stable in summer. However, during winters the enhanced MAV inflow combined with stronger westerly winds and the Mediterranean Outflow Water (MOW) outflow and may cause the vanishing of the EAG and the eastward migration of the WAG (Heburn and La Violette, 1990).

Below the MAW, between ~220 and 1100 m water depth, the Levantine Intermediate Water (LIW), a water mass generated in the eastern Mediterranean Sea flows (La Violette, 1986). This water mass has a relatively constant temperature around ~13.1–13.2 °C and salinity of ~38.5 (e.g., Millot et al., 2006). The deeper part of the Alboran Sea is filled with the eastern Mediterranean Deep Water (WMDW). The LIV and the MOV together form the MOW, which flows through the Gibraltar Strait to reach the Rockall Channel in the North Atlantic (Iorga and Lozier, 1999). The Western Mediterranean Deep Water (WMDW) forms in the Gulf of Lion: it has a temperature of ~12.8–12.9 °C and salinities reaching values of ~38.42–38.45 in the western Mediterranean sub-basins (van Haren and Millot, 2004). This water mass contributes to the regional circulation patten and to the density of the MAW and the LIW (Cacho et al., 2000).

The Alboran Sea is the more eutrophic part of Mediterranean Sea, which is otherwise oligotrophic. Primary production values may range between 215 and 250 g C m^−2^yr^−1^ in the Alboran Sea (Antoine et al., 1995; Bosc et al., 2004). Upwelling of nutrient-rich waters off Malaga and along the Almeria-Oran Front are driven by westerly winds in the norther part of the WAG (e.g., Sarhan et al., 2000; Fabres et al., 2002; van Wambeke et al., 2004). According to van Rooji et al. (2013) and Stalder et al. (2018) the sites investigated in this study are today located within the LIW with temperature and salinity around 13.1 °C and 38.4, respectively and with concentration of dissolved oxygen of ~164–169 μmol/kg in summer.

## 3 Material and methods

**Figure 2:**
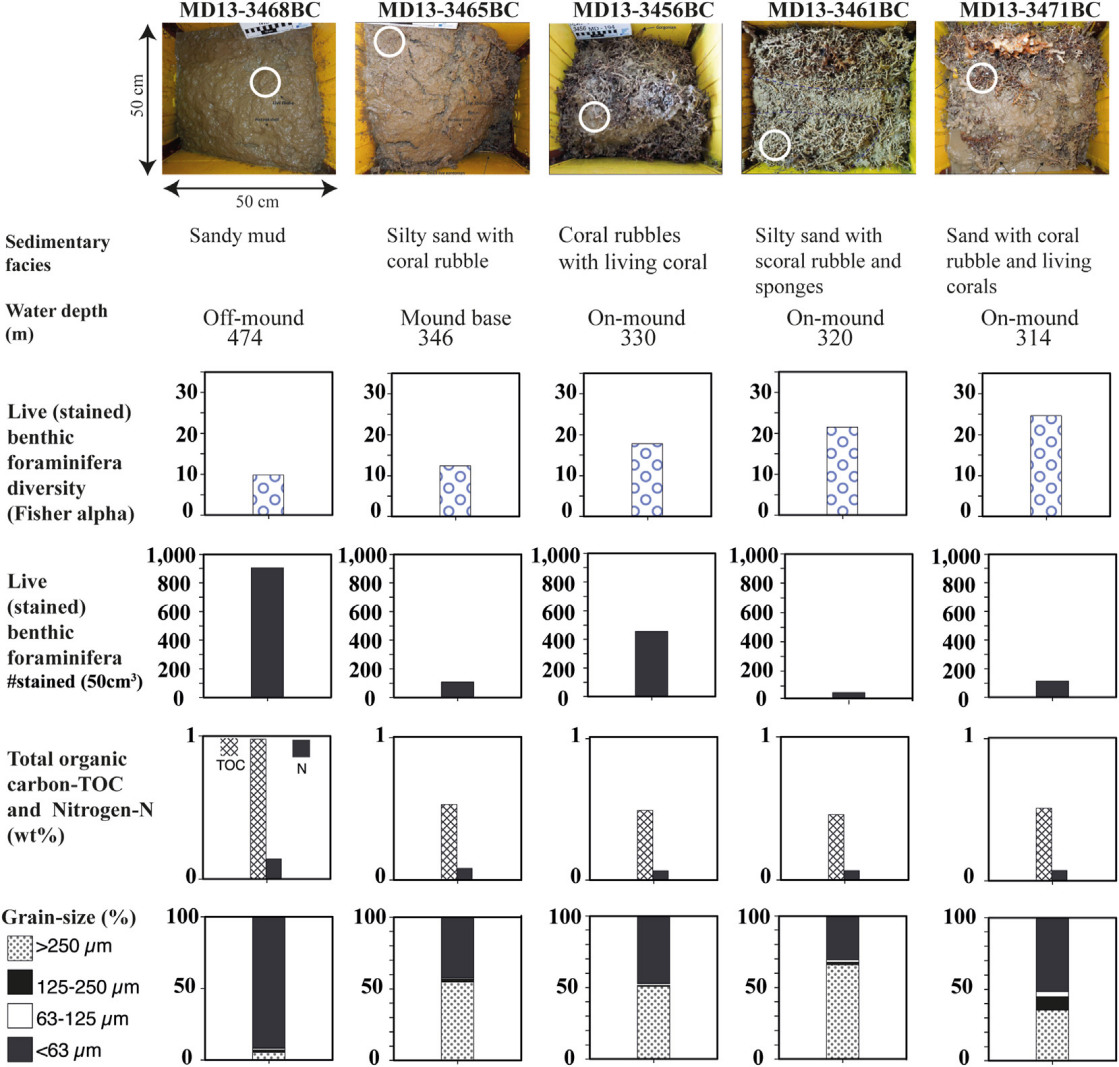
Box core surfaces (0–1 cm) and sedimentary facies and respective water depths. White circles indicate the position of samples for micropaleontology and geochemistry. Shown are the diversity (Fisher alpha index) of live (stained) benthic foraminifera, their number of live (stained) per volume of 50 cm^3^, total organic carbon (TOC), total nitrogen (N) and grain-size.

We investigated five box cores recovered during the Eurofleets Marion Dufresne 194 cruise in June 2013 in the eastern Alboran Sea at water depths ranging from 251 m to 474 m (Figs. 1A-B; Table S1). Two box cores were collected on the top of the Banc des Provençaux "Brittlestar Ridge I" (MD13-3461BC and MD13-3456BC) and one at its western base (MD13-3465BC). One box core (MD13-3468BC) was collected ~300 m SW of the Brittlestar Ridge I (Figure 1B). A fifth box core (MD13-3471BC) was collected on the Cabliers Bank. The box cores were photographed and described on board for living fauna, sedimentary facies and structures (van Rooji et al., 2013) (Figure 2). A CTD cast deployed southeast of the "Brittlestar Ridge I″ down to a water depth of 372 m provided temperature, dissolved oxygen and salinity data throughout the water column (van Rooji et al., 2013; Stalder et al., 2018).

Until now up to 12 sedimentary facies and related models of CWC mounds and reefs based on ROV and video survey data have been proposed (e.g., Freiwald et al., 2002; Foubert et al., 2005; Dorschel et al., 2007; Rosso et al., 2010; Vertino et al., 2015). To describe the box core surface with the best possible detail, the classification of Schönfeld et al. (2011) is applied.

**Figure 3:**
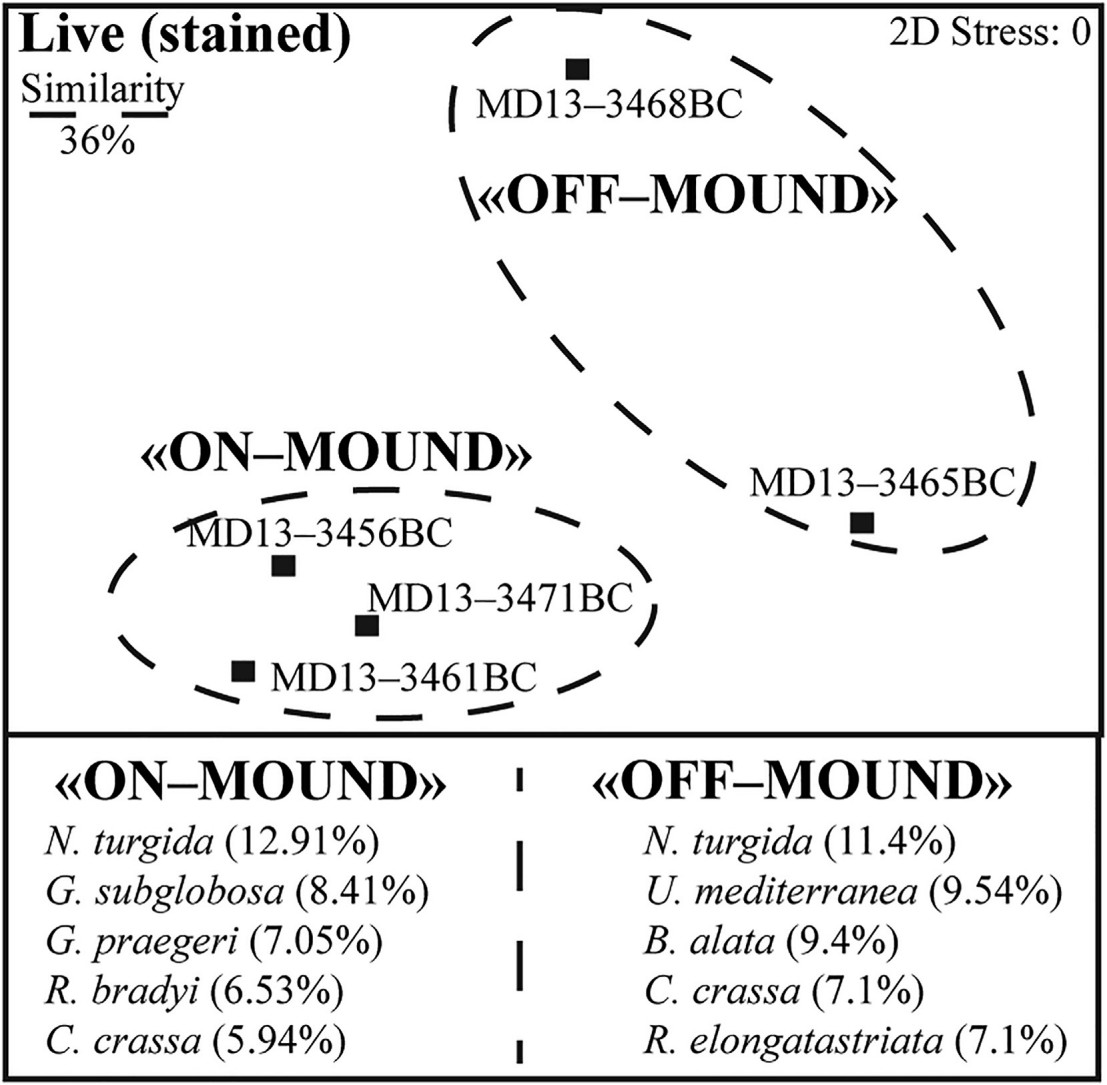
Non-metrical multidimensional scaling (nMDS) plot. The two (stained) benthic foraminiferal assemblages separate at 36% of similarity: the off-mound and the on-mound assemblages, major species and their contribution to the total similarity.

The standard FOBIMO protocol of Schönfeld et al. (2012) was applied for sampling and processing the samples for foraminiferal investigations. Samples were collected with a 8-cm diameter ring (corresponding to around 50 cm^2^ of sediments; Figure 2). The first cm of sediments were treated onboard in an ethanol/rose Bengal solution (1L/2 g of rose Bengal). In laboratory, they were washed through the >63 μm mesh sieve and investigated for their foraminiferal content. If the residues contained more than 300 specimens, they were split with a dry splitter. If the residue contained less than 300 specimens, all specimens were counted. They were identified, counted and glued on plummer-cells and archived (Table S2). They are stored at the Department of Geosciences-Earth Sciences of the University of Fribourg. Most of the box core surfaces contained dense biogenic fragments and they were also investigated for attached epibenthic foraminifera, that, if present, were included in the counting. Quantitative data of benthic foraminifera were statistically treated with the Software PRIMER 6 (Clarke and Gorley, 2006) to calculate the Fisher alpha Index and to obtain the non-Metrical Multi Dimensional Scaling-nMDS (Figure 3).

Carbon and nitrogen stable isotope composition (*δ*^13^C_org_ and *δ*^15^N_org_) of the organic fraction in sediment samples was separated using acid (10% HCl). The δ^13^C_org_ and δ^15^N_org_ were determined by continuous flow elemental analysis/isotope ratio mass spectrometry (EA/IRMS). The system consists of a Carlo Erba 1108 elemental analyzer (Fisons Instruments, Milan, Italy) connected to a Delta V Plus isotope ratio mass spectrometer via a ConFlo III split interface (both instruments from Thermo Fisher Scientific, Bremen, Germany). The standard used for carbon is the Vienna Pee Dee Belemnite (VPDB) and for nitrogen atmospheric nitrogen (Air–N_2_). The calibration and assessment of reproducibility and accuracy of the stable isotope analysis were based on replicate analyses of laboratory standards and international reference materials. Reproducibility and accuracy were better than ±0.1‰ for *δ*^13^C_org_ and ±0.2‰ for *δ*^15^N_org_. The total nitrogen concentration (N_total_ in wt.%) in the HCl-treated sediment samples was determined from the peak areas of the major isotopes using the calibration for *δ*^15^N_org_. The reproducibility was better than 0.2 wt.%.

Total organic carbon content (TOC, in wt.%) was analysed on about 100 mg bulk sediment using the Rock-Eval6 following the standard rock pyrolysis (Espitalié et al., 1986; Bé har et al., 2001). The Hydrogen Index (HI), expressed in mg HC/g TOC, displays the total amount of pyrolyzed hydrocarbons resulting from the cracking of non-volatile organic matter (HI = S2x100/TOC) and the Oxygen Index (OI, in mg CO^2^/g TOC), which accounts for the amount of CO^2^ generated during the pyrolysis of the kerogen (OI = S3x100/TOC), both normalized to TOC. The Mineral Carbon (MINC) represents the percent of carbon derived from inorganic sources. All geochemical analyses were performed at the University of Lausanne and are detailed in Table S2.

## 4 Results

### 4.1 Sedimentary facies and macrofauna

The surfaces of the investigated box cores are characterized by different sedimentary facies (Figure 2). In particular, the surface of MD13-3468BC consists of brownish sandy mud with very rare living macrofauna (mostly feather-duster worms and ophiuroids), barren of CWC debris, typical for off-mound facies. MD13-3465BC, collected at the base of the Brittlestar Ridge (Figure 1B), is characterized by silty sand and biogenic fragments, mostly scleractinians (*M. oculata, D. pertusum, D. dianthus, Dendrophyllia cornigera)* and secondarily molluscs and brachiopods. The coral fragments, moderately to heavily black-coated, are partially covered by a thin brownish silty sand veneer. The exposed skeletal parts are bioeroded and colonised by sessile and vagile epifauna, among which tiny colonies of acanthogorgiid octocorals, sponges, scyphozoans, actinians, bryozoans, serpulids, molluscs and tiny brachiopods (*Terebratulina retusa)*. The surface sediment of MD13-3465BC, characterized by sand and coral fragments, is herein attributed to the transitional facies "buried corals at the mound base" sensu Schönfeld et al. (2011). As typical of on-mound facies, the surface of MD13-3456BC, MD13-3461BC and MD13-3471BC (Figure 2) mostly consists of coral rubble (with broken scleractinian branches up to 20 cm long) intermingled with biogenic-rich silty sand. The surface sediment of MD13-3461BC is characterized by both black-coated and fresh-looking fragments of *M. oculata* and secondarily *D. cornigera*. These coral fragments are only partly colonised by living sessile endo- and epifauna (mostly tiny sponges and erect bryozoans) and also the vagile fauna is scarce (mostly sparse ophiuroids). The MD13-3461BC surface displays strong similarities with the "silty sand with coral rubble and sponges" facies *sensu* Schönfeld et al. (2011). The surface of MD13-3456BC hosts more abundant black-coated coral fragments (almost exclusively *M. oculata* and *D. pertusum*) colonised by a relatively diverse and common living benthic macrofauna, including rare corals (*D. dianthus* and acanthogorgiid ocotocorals), typical of the "coral rubble with living coral" facies. Live sessile and vagile macrobenthos is particularly abundant and much more diverse at the surface of MD13-3471BC where several tiny live *D. dianthus* and a small broken colony of *D. cornigera* occur (Figure 2). Diversified sponges and bryozoans are particularly common, but also tunicates, actinians and sessile brachiopods and molluscs are rather common. This sample is attributed to the "sand with coral rubble and living coral facies".

### 4.2 Live (stained) benthic foraminifera

**Figure 4:**
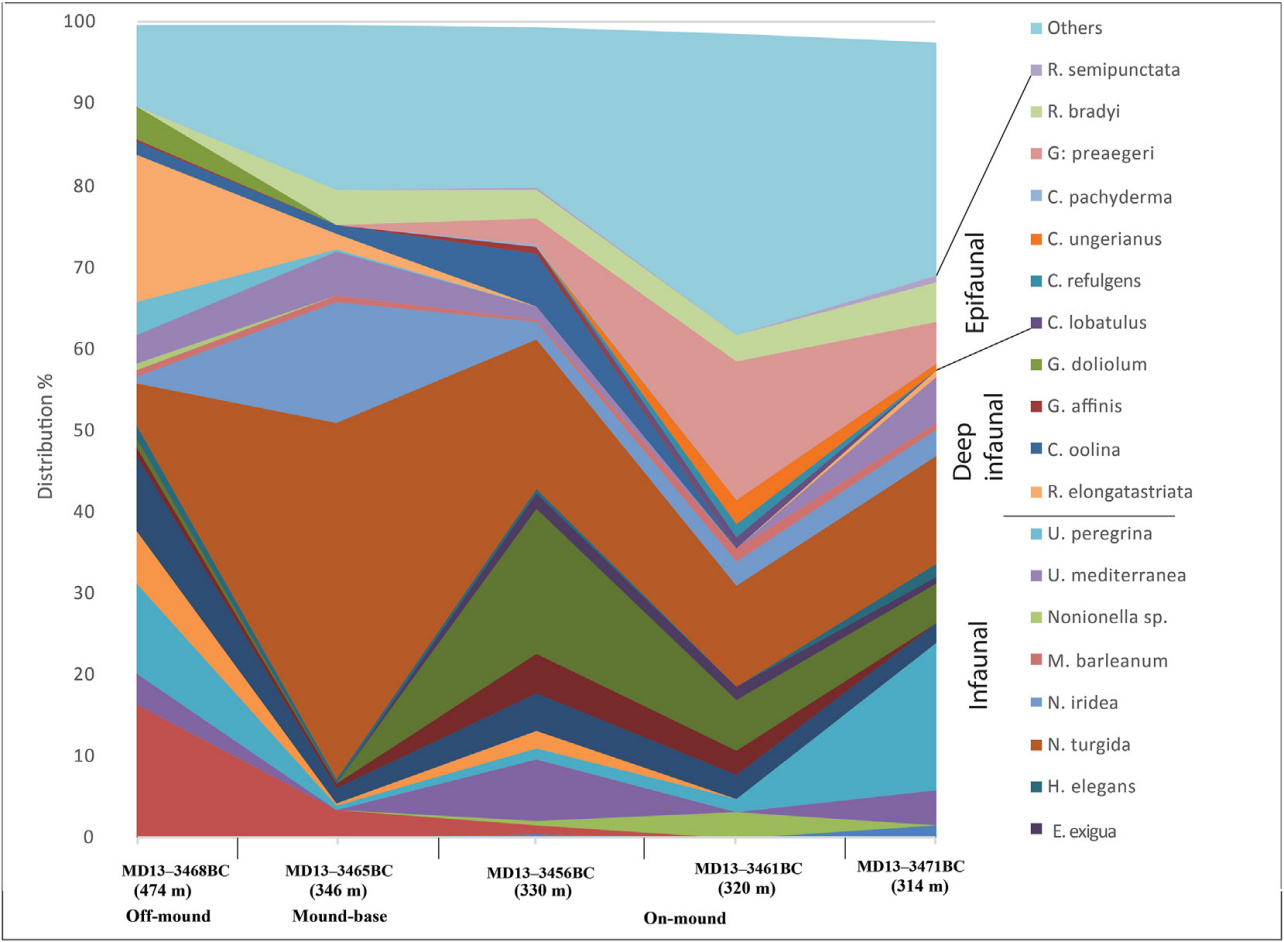
Distribution (in % of total fauna) of selected live (stained) benthic foraminifera in the investigated surface samples.

**Figure 5:**
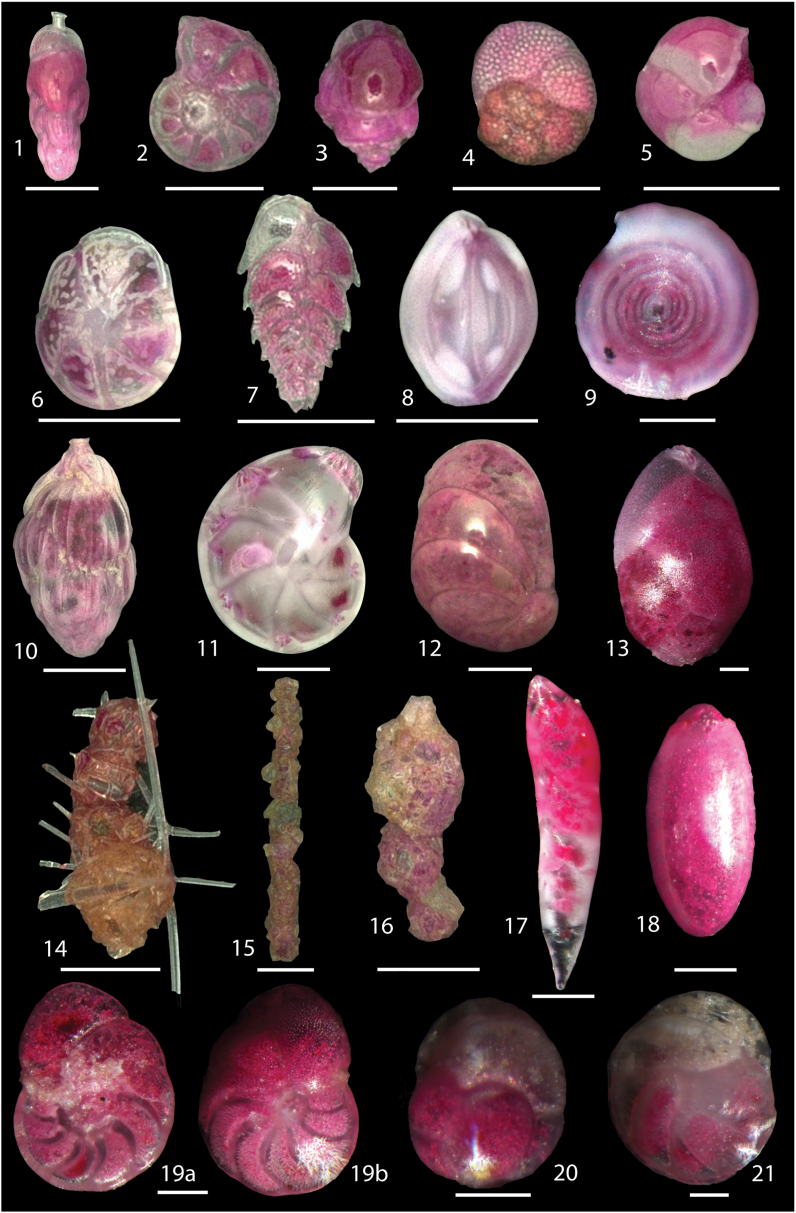
Digital images of selected live (stained) benthic foraminifera from surface sediments (0–1 cm). **1.***Rectuvigerina elongatastriata* Colom, 1952; **2.***Hyalinea balthica* (Schröter in Gmelin, 1791); **3.***Bulimina marginata* d’Orbigny, 1826; **4.***Rosalina bradyi* (Cushman, 1915); **5.***Pullenia quadriloba* Reuss, 1867; **6.***Hoeglundina elegans* (d’Orbigny, 1826); **7.***Bolivina alata* Seguenza, 1862; **8.***Quinqueloculina cuvieriana* d’Orbigny, 1839; **9.***Cornuspira involvens* (Reuss, 1850); **10.***Uvigerina mediterranea* Hofker, 1932; **11.***Lenticulina suborbicularis* Parr, 1950;**12.***Cancris auriculus* (Fichtel and Moll, 1798); **13.***Globobulimina doliolum* (Terquem and Terquem, 1886) **14.***Bigenerina nodosaria* d’Orbigny, 1826; **15.***Rhabdammina abyssorum* Sars, 1869; **16.***Reophax scorpiurus* Montfort, 1808; **17.***Laevidentalina* sp.; **18.***Chilostomella oolina* Schwager, 1878 **19a-b**; *Discorbinella berthelothi* (d’Orbigny, 1839). **20.***Globocassidulina subglobosa* (Brady, 1881); **21.***Cassidulina carinata* Silvestri, 1896. Figures 1–8, 10–12, 14–16 = 250 μm; Figures 9, 13, 17–18; 19a–b = 100 μm; Figure 20–21 = 50 μm.

One-hundred and two (102) live (stained) benthic foraminifera species attributed to 70 genera were identified in surface sediments of the MMF and Cabliers Sites (Table S2). The distribution of common (>3% of the total fauna) live (stained) benthic foraminifera is given in Figure 2 and the abundances of selected species are represented in Figure 4. Some selected species are also documented in Figure 5. Only *Nonionella turgida* is frequent in all box cores contributing between 4.9 to 43.1% to the total live fauna. However, no clear distribution pattern of this species is identified. The infaunal *Nonionella iridea* is abundant only at the mound-base characterized by highest *δ*^13^C_org_ and *δ*^15^N_org_ values (Figure 2). The epibenthic *Gavelinopsis praegeri* (up to 16.9%) and the infaunal *Globocassidulina subglobosa* (up to 17.9%) are frequent on-mound but are absent off-mound and at the mound-base (Figure 4). The epibenthic *Rosalina bradyi* is most abundant on-mound (up to 11%. MD13-3456BC) and is absent in fine pelagic off-mound sediments. Similarly, the epibenthic *Cibicides ungerianus*, *Gyroidina lamarckiana* and *Spirillina vivipara* are absent off-mound and rare at the mound-base.

*Cassidulina crassa* and *C. laevigata* are relatively more abundant (4.49 and 9.2% of the total assemblages, respectively) off-mound sediments and in box core MD13-3456BC whereas, they occur with insignificant abundances at the mound-base, the mound-top and at the Cabliers site. A similar trend can be recognized for the deep infaunal *B. marginata* and the infaunal *Bolivina dilatata*. A relatively weak co-occurrence of these two species (Table S2) in fine grained sediments with higher TOC/N_total_ can be observed (Figure 2). *Bolivinella striatula* is a dominant taxon at the Cabliers site and off-mound, which are mostly characterized by fine sediments (Figure 2 and Table S2).

The infaunal *Bolivina alata* and *Rectuvigerina elongatastriata* (Figure 4) are abundant in the surrounding off-mound sediments and at the mound base with contributions of up to 16.3% and 18.1% respectively. Both species become rare or not present in the live benthic foraminiferal fauna of the on-mound sediments (Figure 4). Similarly, the shallow- *Uvigerina peregrina* and the deep-infaunal *Globobulimina doliolum* and the rarer *Globobulimina affinis* are found off-mound and at the mound-base. They show a relatively good correlation to TOC and to sediment surfaces free of large CWC and other biogenic fragments (Figure 2). *Uvigerina mediterranea* (Figure 4) shows a relatively low contribution to the living assemblage with highest values at the mound-base and the Cabliers site (up to 5.7%). This species is extremely rare on the Brittlestar Ridge I with a contribution of 1.6% in MD13-3456BC and is absent in MD13-3461BC (Figure 4).

Population densities vary from 65 to 608 living specimens per 50 cm^3^ (Figure 2). The fine-grained off-mound sediments contained the highest population density followed by the two box cores hosting high macrobenthic diversity and living CWC corals. Box core MD13-3461BC is characterized by relatively high diversity but low density in foraminifera. Species richness (SR) is high in MD13-3456BC (52 species) followed by MD13-3471BC (44 species), MD13-3468BC (41 species), MD13-3465BC (36 species) and MD13-3461BC (30 species). The diversity index Fisher alpha (Figure 2) of living benthic foraminifera (9.9–24.7) shows a negative correlation to water depth and higher scores in the on-mound samples (Figure 2). The highest Fisher alpha (24.7) of living assemblages is recorded in MD13-3471BC.

### 4.3 Geochemical characterisation

The TOC values range from 0.46 to 0.94 wt.% and display highest values in the off-mound sample and fairly similar values in all other samples (Figure 2). The total nitrogen (N_total_) with values between 0.08 and 0.14 wt.% follows similar trends as the TOC. In general, both the TOC and the N_total_ show a positive correlation to fine-grained sediments (Figure 2). The molar ratio of TOC/N_total_ ranges from 7.24 in MD13-3465BC to 8.33 in MD13-3456BC (Figure 2).

The stable carbon and nitrogen isotope compositions of the organic fraction show values between -24.1 and -22.1‰ for *δ*^13^C_org_ and 2.0 and 5.3‰ for *δ*^15^N_org_ (Figure 2). The *δ*^13^C_org_ values are significantly correlated with the *δ*^15^N_org_ (r = 0.88). The on-mound samples display generally lower *δ*^13^C_org_ and *δ*^15^N_org_ than the off-mound and mound-base samples except for MD13-3471BC (Figure 2). Although there is no straightforward relation between the lower *δ*^13^C_org_ and *δ*^15^N_org_ values and the grain-size, the samples with coarser sediments are generally depleted in ^13^C and ^15^N values compared to the finer ones (Figure 2).

### 4.4 Statistical treatment

The non-metric Multidimensional Scaling (nMDS) (Figure 3) provides useful information about discrete distribution patterns of foraminiferal assemblages and the contribution of each species to the total similarity (Clarke and Gorley, 2006). Two assemblages separate in the nMDS: the "on-mound" and the "off-mound" (Figure 3). Nineteen species account for 90.56% of the average similarity of the "on-mound" assemblage. Dominant species are *N. turgida*, *G. subglobosa*, *G. praegeri*, *R. bradyi* and *Cassidulina crassa* (Figure 4; Table S2). For the "off-mound" assemblage, seventeen species contribute 92.89% of the average similarity. The major contributors of the assemblage are *N. turgida*, *U. mediterranea*, *B. alata*, *C. crassa* and *R. elongatastriata* (Figure 4; Table S2). The samples from the "on-mound" assemblage are characterized by the relatively high abundance of CWC fragments. The samples plotting into the "off-mound" assemblage are from the surrounding pelagic sediment (MD13-3468BC) and from the mound base (MD13-3465BC), devoid of CWC fragments or partly covered by a pelagic sediment layer.

## 5 Discussion

Video survey of the MMF showed that most of the elongated ridges occurring in this area (e.g., Comas et al., 2009), mostly consist of dead coral framework with abundant coral rubbles and that small living CWC colonies are presently restricted to their tops (Hebbeln et al., 2009). Today a fine sediment blanket covering the dense coral rubble at the mound base together with a live benthic foraminiferal fauna comparable to the surrounding pelagic sediments suggest that the MMF is exposed to siltation. According to Lo Iacono et al. (2014), the burial of the MMF started in the late Holocene and sediment transport is still active in this area.

However, the reason why sparse surviving CWCs still occur on top of ridges has not been explored. Should the quality and availability of organic matter (OM) and/or other specific hydrographic conditions account for the presence of these “survivors”?

### 5.1 Autoecology of benthic foraminifera

The autoecology of the most abundant species identified in this research is summarized in Table 1. Living assemblages include species that occur today in the Mediterranean Sea (e.g., Margreth et al., 2011; Martins et al., 2016; Stalder et al., 2015; 2018). In the investigated off-mound/mound-base assemblages the overall large abundances of opportunistic infaunal taxa such as *N. turgida*, *R. elongatastriata, Bolivina striatula,* bolivinids, and cassidulinids can be linked to the elevated abundances of relatively fresh OM exported during the spring bloom (see Table 1 for summary of references). In particular, this is clearly shown by dominant *N. turgida*, which contributes up to 43.1% in box core MD13-3465BC. This species shows highest abundances during the eutrophic spring bloom conditions of April–May along the Rhône River prodelta when large amount of fresh OM reaches the seafloor (Goineau et al., 2012). An assemblage dominated by intermediate to deep infaunal species such as *R. elongatastriata*, *M. barleeanum*, *C. oolina* and bolivinids has been reported from the Saint-Tropez canyon (Bay of Fréjus) and associated to fine grained sediments enriched in OM (Fontanier et al., 2008). The same authors associate the relatively high diversity to a specialized climax community able to colonize habitats enriched in OM with variable dysoxic to anoxic conditions. We interpret the distribution of intermediate to deep infaunal species within the first centimetre of sediments as an opportunistic strategy in response to high availability of labile OM at the water-sediment interface. This is well in agreement with previous observations on vertical migration of certain intermediate to deep infaunal species within surface sediment layers (e.g., Ernst et al., 2000; Koho et al., 2008, 2011).

**Table 1:** Autoecology of the most important species identified in this research, their living strategy, preferred substratum and other ecological characteristic according to the literature.

Species	Living strategy	Preferred substratum	Feeding strategy	Oxygen	Energy	Other ecological preferences	References
*Astrononion galloway*	Infaunal	Mud to sand	-	-	High energy	-	See Spezzaferri et al. (2015), and reference therein
*Bolivina alata*	Shallow infauna	Mud	-	Can tolerate low oxygen	Low energy	Adapted to meso-eutrophia	Murray (1991); Schmiedl et al. (2003); Stefanelli et al. (2005); Drina et al. (2007)
*Bolivina dilatata*	Shallow infauna	Mud	-	Can tolerate low oxygen	Low energy	Adapted to meso-eutrophia	Stefanelli et al. (2005)
*Bolivina difformis*	Shallow infaunal	Coarse sand	-	Can tolerate low oxygen	-	-	See Spezzaferri et al. (2015), and reference therein
*Bolivinella striatula*	Infaunal	Mud	Prefers high- quality OM proteins enriched, carbohydrates and chlorophyll-α.	Tolerates low oxygen	-	-	Murray (2006); Dimiza et al. (2019); Martins et al. (2016)
*Bulimina marginata*	Deep infaunal in anoxic sediments, also shallow infaunal	Mud, silt	May feed on low- quality OM	Can tolerate dysoxia-anoxia	Low energy	Prefers high productivity	See Spezzaferri et al. (2015), and reference therein
*Cassidulina crassa*	Infaunal	Mud	Prefers High-quality OM	Tolerates low oxygen	-	-	This research
*Cassidulina laevigata*	Infaunal	Mud to sand	Prefers high quality OM	Tolerates low oxygen	-	Opportunistic, moderate to high carbon flux rates	See Margreth et al. (2009) and reference therein
*Chilostomella oolina*	Intermediate to deep infaunal	Mud to silt	Prefers high amounts of OM, positively reacts to labile OM input	Tolerates anoxia and dysoxia	-	Organic matter rich sediments	Langezal et al. (2006)
*Globobulimina doliolum*	Intermediate to deep infaunal	Mud	Can feed on low and intermediate OM	Tolerates anoxia and dysoxia	-	Organic matter rich sediments	This research
*Globobulimina affinis*	Intermediate to deep infaunal	Mud	Can feed on low and intermediate OM	Tolerates anoxia and dysoxia	-	Organic matter rich sediments	See Spezzaferri et al. (2015), and reference therein
*Globocassidulina subglobosa*	Infaunal	Mud	Phytodetritus feeder, preferentially fresh diatoms	-	-	Prefers oligothophy	See Spezzaferri et al. (2015), and reference therein
*Hoeglundina elegans*	Shallow infaunal	Mud and silt	Prefers high- quality OM	Oxic with limited tolerance to low oxygen	-	Prefers oligothophy	See Spezzaferri et al. (2015), and reference therein
*Melonis barleanum*	Intermediate infaunal	Mud to silt	High OM	-	-	Prefers temperatures <10 °C, lives in high productiv waters	See Spezzaferri et al. (2015), and reference therein
*Nonionella turgida*	Infaunal	Mud		Tolerates suboxia and anoxia	-	-	See Spezzaferri et al. (2015), and reference therein
*Nonionella iridea*	Infaunal	Mud	Positively reacts to phytoplankton bloom. High- quality OM	Tolerates suboxia and dysoxia	-	Opportunistic	See Margreth et al. (2009) and reference therein
*Rectuvugerina elongatastriata*	Intermediate to deep infaunal	Mud to silty sand	Prefers high- quality OM	-	-	Prefers eutrophy, stenohaline	Fontanier et al. (2008); Avnaim-Katav et al. (2020);
*Uvigerina mediterranea*	Shallow infaunal	Mud	Rich supply of labile OM	Oxic less tolerant to suboxia than *U. peregrina*	-	Prefers eutrophy	See Spezzaferri et al. (2015), and reference therein
*Uvigerina peregrina*	Shallow infaunal	Mud	Rich supply of labile OM	Tolerates suboxia	-	Prefers eutrophy	See Spezzaferri et al. (2015), and reference therein
*Cibicides refulgens*	Epifaunal attached	Hard substratum	Passive suspension feeder, predator	Oxic	High energy	Stable physico-chemical conditions	See Spezzaferri et al. (2015), and reference therein
*Cibicides ungerianus*	Epifaunal attached, shallow infaunal	Mud	Passive suspension feeder	Oxic	High energy	Stablephysicochemical conditions	See Margreth et al. (2009) and reference therein
*Epistominella exigua*	Epifaunal-shallow infaunal	Mud	Phytodetritus feeder, positively reacts to seasonal food fluxes	-	-	Large tolerance to varying organic flux	See Margreth et al. (2009) and reference therein
*Gavelinopsis praegeri*	Epifauna attached, mobile	Hard substratum	Suspension feeder	Well oxygenated waters	High energy	-	See Spezzaferri et al. (2015), and reference therein
*Rosalina bradyi*	Epifaunal attached	Hard and/or coarse substratum	-	Can tolerate drastic oxygen depletion	-	Can tolerate drastic salinity changes: euryhaline	Fontanier et al. (2008); Stalder et al. (2018)
*Rosalina semipunctata*	Epifaunal attached	Hard substratum	-	Well oxygenated waters	High energy	-	Hawkes and Scott (2005)

The on-mound assemblage differs from the off-mound one by its relatively important contribution of epibenthic species such as *C. refulgens*, *C. ungerianus*, *G. praegeri*, *R. bradyi* and *R. semipunctata,* the rare occurrence of *Gyroidina lamarckiana*, *Gyroidina altiformis* and/or epifaunal to shallow infaunal species such as *E. exigua*. Most of these species are exclusively found on-mound and/or rare to absent at the mound-base and thus their occurrence can be linked to the availability of elevated hard substrates and specialized microhabitats below a dense biogenic cover. (e.g., CWC debris) at the seafloor.

The absence of the epifaunal specie *Discanomalina coronata* considered as a bioindicator of CWC by Margreth et al. (2009, 2011), Spezzaferri et al., 2013, Spezzaferri et al., 2015 and Stalder et al. (2015) is possibly due to the declining of CWC in the investigated region, where ecological conditions do not support anymore the life of this species, like they do in active CWC settings, such as the Norwegian margin (e.g., Spezzaferri et al., 2013).

The on-mound much higher diversity suggests that this elevated substratum coupled with finer sediment between coral branches offers more ecological niches and microhabitats for more diverse epifaunal and infaunal benthic foraminifera assemblages, although sometimes specimen density may be low, which corresponds well to the conclusions of Margreth et al. (2009) and Schönfeld et al. (2011).

### 5.2 Geochemical characterization

**Figure 6:**
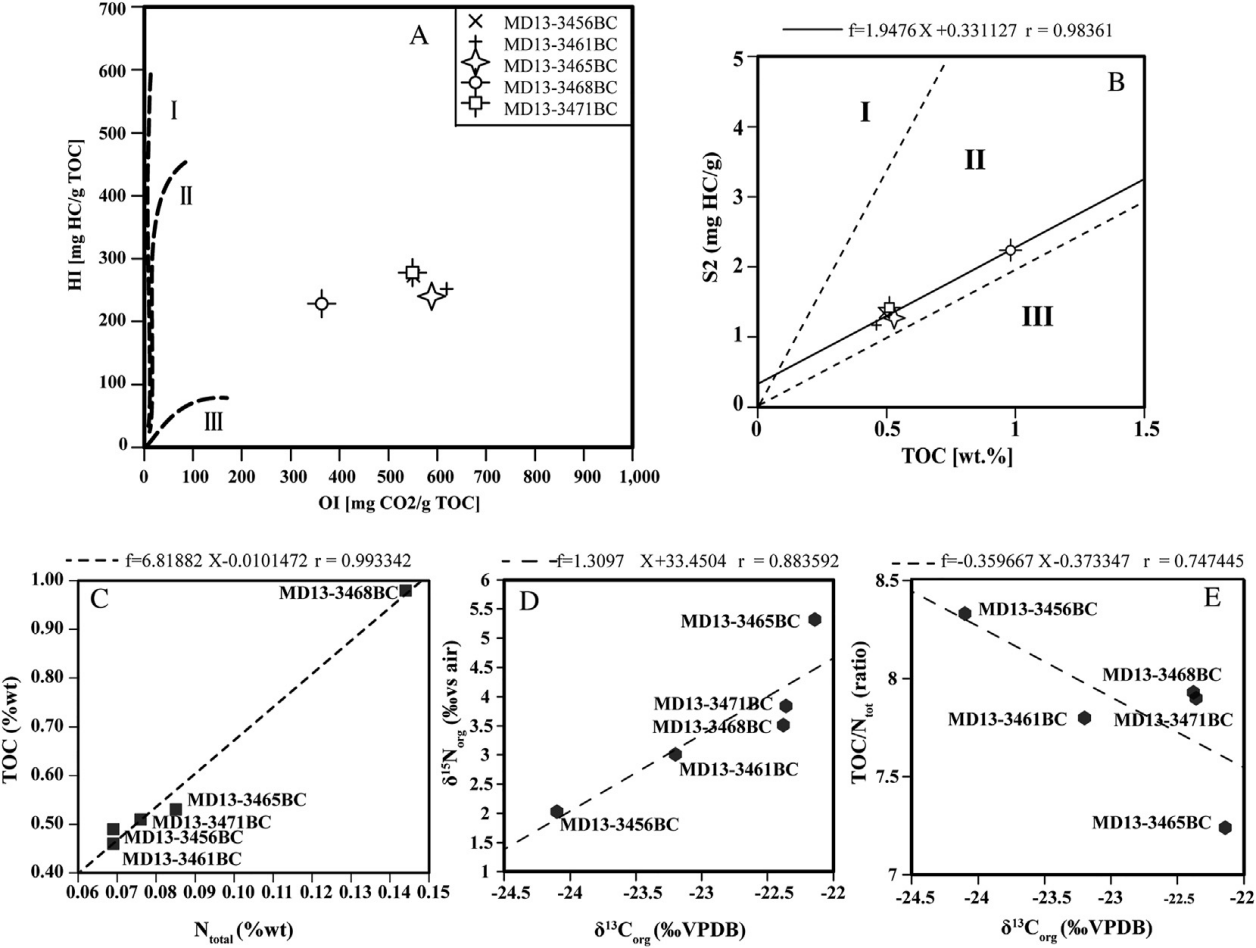
A, B. Pseudo Van-Krevelen plot Individual S2 versus TOC plots. Boundaries of the kerogen typed fields are according to eron Langford and Blanc-Vall (1990). C-E. Plots showing the sedimentary organic matter characterization, from left to right: C. TOC versus N; D. *δ*^15^N_org_ versus *δ*^13^C_org_ versus and E. TOC/N_total_ (molar ratio) versus *δ*^13^C_org_.

The regression line of the sediment plots within type II kerogen field, indicates that the OM in the studied region has a dominant marine origin. The calculated constant HI (194 mg HC/g TOC) from the slope of the regression line and the excellent correlation coefficient (*r* = 0.98) confirm a common source of predominantly marine organic matter in all samples (Fig. 6A-B). This marine origin is also supported by the TOC/N_total_ ratios and the slope-derived TOC/N_total_ value of 6.81 (Figure 6C). The presence of significant amounts of inorganic N in the sediments can be ruled out as the intercept is close to the axes origin (Figure 6C).

Marine OM is generally isotopically heavier than terrestrial OM (Schubert and Calvert, 2001). Therefore, the *δ*^13^C_org_ values (Figure 6D), in particular in MD13-3456BC and 3461BC (-24.10 and -23.20%, respectively) suggest also that some of the OM may be a mixture of marine and older reworked particulate organic matter, compared to typical values from temperate phytoplankton (ranging from -22.0 to -19.0‰, Hedges et al., 1997). Both samples display the lowest *δ*^15^N_org_ values (Figure 6D) that can be related to the presence of terrigenous OM components characterized by depleted *δ*^15^N_org_ as reported from the Tay Estuary in Scotland (Thornton and McManus, 1994) and from the Gulf of Trieste in the Adriatic Sea (Ogrinc et al., 2005). Indeed, Terhzaz et al. (2018) show that sediments from the Alboran Sea contain also some components from the Moroccan hinterland transported into the sea by winds and rivers and redistributed by bottom currents.

The nitrogen isotope composition of organic matter depends on the type and origin of dissolved inorganic nitrogen and numerous biologically mediated reactions, such as fixation, assimilation, mineralisation (ammonification), nitrification, denitrification and anaerobic ammonium oxidation (anammox). The sedimentary organic nitrogen would also be affected by mineralization under different redox-conditions (oxygen content), heterotrophic reworking and sediment transport. Pantoja et al. (2002) have estimated to 2.1 ± 1.8‰ the mean *δ*^15^N_org_ value of phytoplankton of Alboran surface waters (<200 m). On their way to the seafloor, suspended particles become enriched in ^15^N through the release of ^15^N-depleted soluble nitrogen during decomposition processes; therefore, sub-surface sediments display generally up to 4‰ higher *δ*^15^N values than at the sea floor (Fry et al., 1991; Altabet and François, 1994; Gaye-Haake et al., 2005). Low *δ*^15^N values (2.03‰) such as in box core MD13-3456BC (Figure 6D) may also be explained by the selective degradation of nitrogen proteins, which will result in a decrease of *δ*^15^N and *δ*^13^C_org_ (Ogrinc et al., 2005 and references therein) or by nitrogen fixation by diazotrophic organisms (Sachs and Repeta, 1999; Zehr et al., 2001). Kerhervé et al. (2001) argued that the low *δ*^15^N of particulate OM in the Gulf of Lions were related to nitrogen fixation by cyanobacteria, and that atmospheric nitrogen fixing may play a crucial role in the Mediterranean Sea. Dachs et al. (1998) also highlight the important contribution of cyanobacterial compounds in sinking particles in the Alboran Sea during springtime and the possible fundamental role they have in the biogeochemical cycle of lipids.

**Figure 7:**
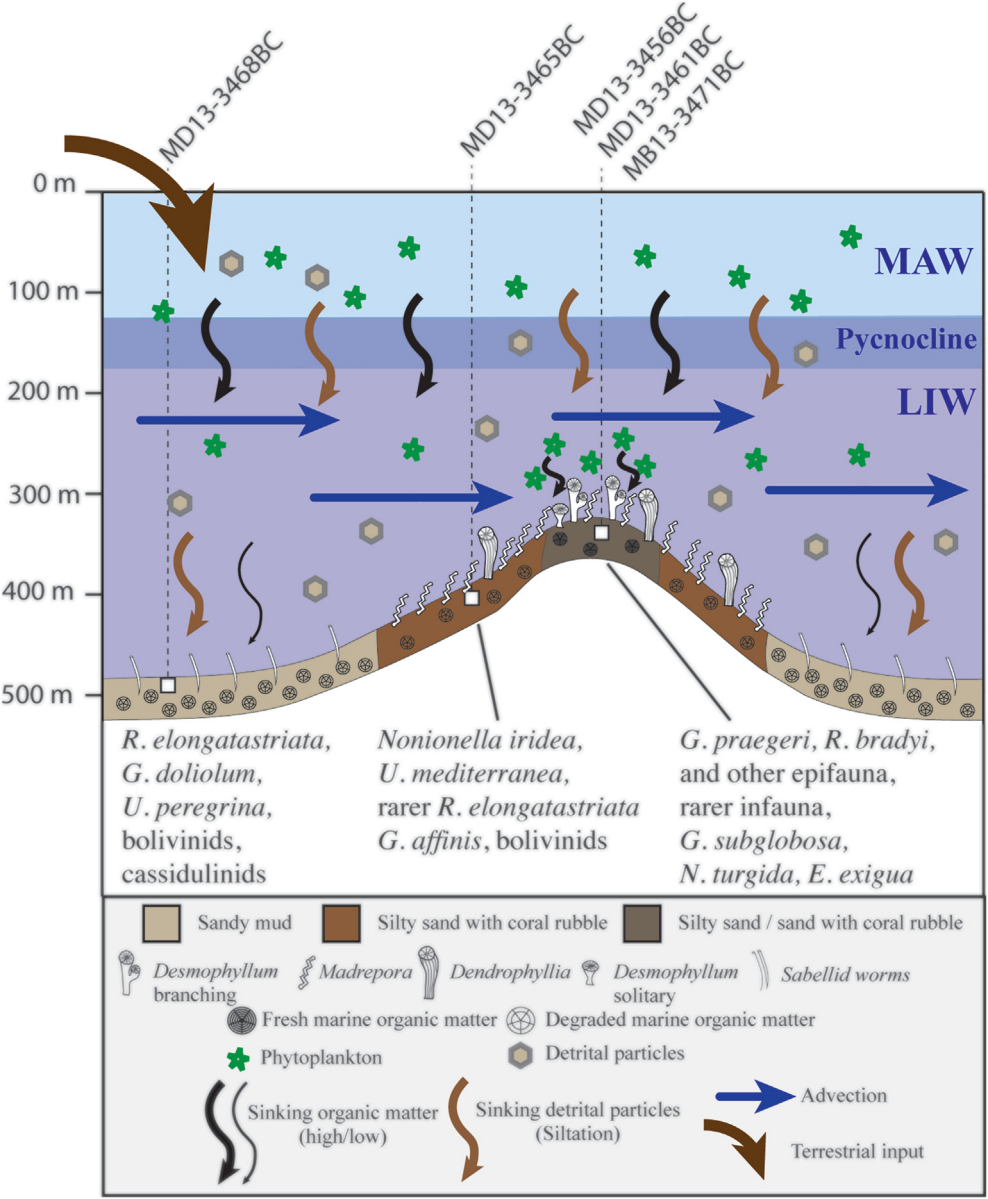
Summary of oceanographic processes occurring at the Brittlestar Ridge I in recent time and dominant benthic foraminiferal assemblages.

Based on our *δ*^15^N and *δ*^13^C_org_ data from surface sediments, we suggest that evident differences of OM degradation and dilution with terrigenous compounds exist among the sites (Figs. 6D-E). It is however difficult to better constrain the extent of the diagenetic control on the nitrogen isotopes. Interestingly, the two samples with the lowest δ^15^N values are located on the upper edge of Brittlestar Ridge I (Figure 1B). Therefore, a more effective advection of freshly exported particulate OM from the surface waters may occur at the mound top rather than at the mound base and off-mound. Such mechanisms providing CWCs with food particles through topographic-induced currents or internal tidal waves have already been demonstrated by Davies et al. (2009) and could partially explain why scarce living CWC colonies still occur on the top of the MMF mounds (Figure 7). Benthic foraminiferal assemblages reflect the input of fresh OM on off-mound with high abundances of high-quality OM preferring-species. The more effective advection of freshly exported particulate OM from the surface waters to the mound top is reflected by the co-occurrence of corals, epibenthic and also infaunal foraminifera thriving with high OM quality (Figure 7).

## 6 Conclusion

The detailed study of benthic foraminifera and CWC fragments in the investigated five box cores provides important information about oceanographic processes occurring today at the sea floor in the eastern Alboran Sea. Our data show that the diversity of benthic foraminifera is higher on-mound because the biogenic fragments at the seafloor offer more numerous microhabitats, from epibenthic to deep infaunal. Geochemical characterization of the surface samples shows that the sediments at the seafloor contain relatively fresh (labile) organic carbon together with reworked refractory material. The *δ*^15^N of the organic fraction strongly suggests that relevant atmospheric N_2_-fixation and degradation processes occur at the MMF and/or that local hydrographic conditions provide the mound-tops with freshly exported phytodetritus and partially explain why living CWC colonies, though scarce, can be still found alive on the top of the MMF mounds.

## Declarations

### Author contribution statement

Claudio Stalder: Conceived and designed the experiments; Performed the experiments; Analyzed and interpreted the data; Wrote the paper.

Akram ElKateb, Loubna Tehzaz, Agostina Vertino: Analyzed and interpreted the data; Wrote the paper.

Jorge E. Spangenberg: Performed the experiments; Analyzed and interpreted the data; Wrote the paper.

Silvia Spezzaferri: Conceived and designed the experiments; Analyzed and interpreted the data; Contributed reagents, materials, analysis tools or data; Wrote the paper.

### Funding statement

The MD194 cruise was funded by the EU FP7th Framework Program under the EUROFLEETS grant Agreement n. 228344.

The Swiss National Science Foundation Grants N. 200020_131829 and 200020_153125 funded the shore based research, and partly funded the ship time.

Stalder Claudio warmly acknowledges the Johanna Resig Cushman Foundation Fellowship.

Spezzaferri Silivia, Loubna Tehzaz and Agostina Vertino thank the ESF COCARDE ERN.

### Data availability statement

Data included in article/supplementary material/referenced in article.

### Declaration of interests statement

The authors declare no conflict of interest.

### Additional information

No additional information is available for this paper.

## Appendix A Supplementary data

Table S1_Coordinates Table S1_Coordinates

Table S2_Quantitativexlsx Table S2_Quantitativexlsx
